# Comparison between the Sofia SARS Antigen FIA Test and the PCR Test in Detection of SARS-CoV-2 Infection

**DOI:** 10.1093/labmed/lmac079

**Published:** 2022-08-17

**Authors:** Manca Černila, Mateja Logar, Hugon Možina, Joško Osredkar

**Affiliations:** Institute of Clinical Chemistry and Biochemistry, University Medical Centre Ljubljana, LjubljanaSlovenia; Faculty of Pharmacy, University of Ljubljana, Ljubljana, Slovenia; Department of Infectious Diseases, Department for Hospital Hygiene, University Medical Centre Ljubljana, Ljubljana, Slovenia; Internist First Aid, University Medical Centre Ljubljana, Ljubljana, Slovenia; Institute of Clinical Chemistry and Biochemistry, University Medical Centre Ljubljana, LjubljanaSlovenia; Faculty of Pharmacy, University of Ljubljana, Ljubljana, Slovenia

**Keywords:** COVID-19, SARS-CoV-2, Sofia SARS antigen FIA, clinical validity, analytical validity, rapid antigen testing

## Abstract

**Objective:**

The purpose of this study was to compare Quidel’s rapid antigen test Sofia SARS antigen Fluorescent Immunoassay (FIA) (Sofia) with the real-time reverse transcription–polymerase chain reaction (rRT-PCR) test.

**Methods:**

Two samples were taken from each test subject—1 for testing with the Sofia test and 1 for testing with the rRT-PCR test. In total, swabs were taken from 146 subjects who presented symptoms of infection (group 1) and 672 subjects who were tested regardless of symptoms (group 2).

**Results:**

In group 1, the sensitivity of the antigen test was 90.0% and its specificity 97.5%. In group 2, however, the sensitivity of the antigen test was 81.4% and the specificity 98.9%. In addition to asymptomatic patients, false-negative results of rapid antigen tests also occurred in subjects with high threshold values (cycle threshold > 30).

**Conclusion:**

Our results show that the Sofia test meets the standards for diagnostic tests according to the criteria of the World Health Organization, as they show high sensitivity and specificity, and perhaps most importantly, a high negative predictive value (> 95%).

Frequent and rapid diagnostic testing is crucial to limit the spread of SARS-CoV-2 in the community, as it allows timely identification and isolation of infected individuals and thus breaks the transmission chain.^1^ The quantitative detection of viral RNA in nasal swab or saliva samples based on the rRT-PCR test is the gold standard for sensitivity in detecting the presence of SARS-CoV-2. However, the lack of reagent supply, significant costs, and infrastructure constraints make it difficult to test sufficiently and report results quickly.^1,2^ These conditions encouraged the development of rapid diagnostic tests, which are based on the detection of viral antigens. Their main advantages lie in the rapid availability of results and the possibility to perform point-of-care testing, which also relieves the burden on staff in diagnostic laboratories. According to the literature, however, the performance of these tests remains uncertain.^3^

Rapid antigen detection kits have so far been described as suboptimally sensitive and specific. Nevertheless, the unique protein domains of the virus can be used to develop kits with higher sensitivity.^4^

Sofia SARS Antigen Fluorescent Immunoassay (FIA) (hereafter Sofia) is a type of antigen test. These tests are designed to detect viral proteins in respiratory samples of persons with COVID-19.^5^ Sofia is an FIA that uses advanced immunofluorescence-based lateral flow technology. It uses the so-called “sandwich method” for qualitative detection of the virus’s nucleocapsid proteins. Sofia, in combination with the Sofia 2 and Sofia analyzers, provides automated and objective results in 15 min, which also allows for the testing of persons with suspected COVID-19 in the person’s immediate environment.^6^ The FIAs are modern fluorescence-based tests that use a fluorescent component (a fluorescent dye-labelled antibody) as a detection reagent.^7^ The Sofia test uses europium in the form of a chelate complex as the fluorescent component for detection.^8^ The wavelength of the excitation light of these complexes is usually about 335 nm, and the wavelength of the emitted light is about 616 nm.^9^ From this data, we can infer the interference caused by molecules that may appear in the sample. Hemoglobin, which absorbs light very efficiently at wavelengths below 600 nm, is the most common potential interferant in samples.^10^ The molecule absorbs light and thus weakens the intensity of the excitation or the emission light of the test. The quenching efficiency also depends on the extinction coefficient of the molecule and its concentration in the sample. This can lead to false-negative results.^11^ Some medicines can cause interference as well, but they are not specifically listed by the manufacturers. The accuracy of the result can be affected not only by interfering molecules that can cause autofluorescence or signal quenching but also by the volume of the sample—insufficient sample volume can give false-negative results.^5^

## Materials and Methods

### Participants and Samples

The experimental use of the Sofia test and its evaluation began in August 2020 at the Department of Infectious Diseases of the University Medical Centre Ljubljana. It was used on all symptomatic patients who were admitted and hospitalized in the grey zone (hereafter referred to as group 1). As the results were satisfactory, the use of the rapid test was extended to other locations of the University Clinical Centre Ljubljana in November. The results were also monitored and collected at the Internist First Aid of the University Medical Centre Ljubljana, where both symptomatic and asymptomatic patients (hereafter group 2) were tested. Prior to collecting the samples, we obtained the permission of the Commission for Medical Ethics (numbers 0120-211/ 2020/7 and 0120-60/ 2021/2), which allowed us to use the data for research purposes if the patient gave their verbal consent.

### Laboratory Analysis

Two samples were taken from each patient using a nasopharyngeal swab. The first sample was used to detect viral antigen using the Sofia test, which was performed at the site of sampling. We used the Sofia SARS antigen FIA (Quidel, San Diego, CA, US) test kit and the fluorimeter SOFIA 2 (Quidel). The “walk away” method was selected in the device, so that the device incubated the plate itself and read the result after 15 min. The result was displayed as + (positive) or − (negative). If the control was valid, that was shown by a green tick mark. Otherwise, the device indicated an error and did not display the test result. In this case, the test must be repeated. The second sample was immersed in 2 to 3 mL virus transport medium and transported to the Institute of Microbiology and Immunology, where it was used for rRT-PCR testing. The transport was made in less than 2 hours and at room temperature.

### Statistical Analysis

For the statistical analysis of the data, we used Excel (Microsoft Corp, Redmond, WA, US) to form contingency tables, the MedCalc Software Ltd statistical program to perform the analyses of sensitivity, specificity, positive and negative predictive values, and accuracy, and finally, the IBM SPSS statistical program was used to compare the tested methods with the McNemar and Kappa tests (α = .05).

## Results

In total, we tested 818 people who were divided into 2 groups. Group 1 included 146 individuals who showed symptoms of the SARS-CoV-2 infection. Group 2, on the other hand, included 674 subjects who were tested regardless of whether they showed any symptoms of infection. In group 1, we only used the results of 132 symptomatic persons in our analysis—3 of the point-of-care testing (POCT) results showed an invalid result and the remaining 11 were excluded due to lack of data. The results of the screening test compared with the gold standard are presented in **TABLE 1**.

**TABLE 1.** Presentation of the Screening Test Results for Group 1 Compared with the Gold Standard

Test Results	Positive Results rRT-PCR n (percentage)	Negative Results rRT-PCR n (percentage)	Total
Positive results Sofia n (percentage)	9 (6.8 %)	3 (2.3 %)	12
Negative results Sofia n (percentage)	1 (0.8 %)	119 (90.1 %)	120
Total	10	122	132

Sofia, Sofia SARS antigen Fluorescent Immunoassay; rRT-PCR, real-time reverse transcription–polymerase chain reaction.

Among the 672 patients in group 2, 2 of the tests were not included in the analysis due to lack of data. Those tests with an invalid result were repeated, as further treatment of patients depended on the result of the tests. The results of the screening test compared with the gold standard are presented in **TABLE 2**.

**TABLE 2.** Presentation of the Screening Test Results for Group 2 Compared with the Gold Standard.

Test Results	Positive Results rRT-PCR n (percentage)	Negative Results rRT-PCR n (percentage)	Total
Positive results Sofia n (percentage)	524 (78.2 %)	6 (0.9 %)	530
Negative results Sofia n (percentage)	29 (3.8 %)	113 (17.0 %)	142
Total	553	119	672

The sensitivity and specificity of the tests were calculated using the data in **Tables 1** and **2** (**Tables 3** and **4**).

**TABLE 3.** Sensitivity, Specificity, Positive and Negative Predictive Values, and Accuracy for Group 1.

Statistic	Value	95% Confidence Interval
Sensitivity	90.00%	55.50%–99.75%
Specificity	97.52%	92.93%–99.49%
Disease Prevalence	7.63%	3.72%–13.59%
Positive Predictive Value	75.00%	49.05%–90.34%
Negative Predictive Value	99.16%	94.84%–99.87%
Accuracy	96.95%	92.34%–99.16%

**TABLE 4.** Calculated Values of Sensitivity, Specificity, Positive and Negative Predictive Values, and Accuracy for Group 2.

Statistic	Value	95% Confidence Interval
Sensitivity	81.43%	73.98%–87.50%
Specificity	98.87%	97.55%–99.58%
Disease Prevalence	20.90%	17.88%–24.17%
Positive Predictive Value	95.00%	89.52%–97.69%
Negative Predictive Value	95.27%	93.44%–96.61%
Accuracy	95.22%	93.32%–96.71%

The results differ between the 2 tested groups. In addition, the values of diagnostic sensitivity and specificity, calculated from our results, also differ from the values stated by the manufacturer. The sensitivity stated by the manufacturer is slightly higher—87.5%, and the specificity is stated as more than 99.9%. It is important to note, that the manufacturer obtained these values when testing frozen samples, so the values of fresh samples may vary. Moreover, we do not have the data on the cycle threshold (Ct) values of the samples tested by the manufacturer.^5^

The values of sensitivity and specificity stated by the manufacturer are high and indicate a good diagnostic reliability of the tests. We observed, however, that the diagnostic reliability of the tests is significantly higher when testing symptomatic persons. The reasons for the differences in test performance may vary. Diagnostic efficiency can be affected, among other things, by the analytical sensitivity of the tests. For this purpose, we analyzed the data further to determine the analytical sensitivity of the tests with the help of the Ct value. In group 1, we only observed 1 false-negative result. The measured Ct value was 15.3, indicating a high concentration of viral RNA in the sample. It is important to note, however, that in this case, the subject had been tested and confirmed positive with the rRT-PCR test 6 weeks before the Sofia test was performed. The negative result was therefore most likely due to the low concentration of the virus in the upper respiratory tract as the disease had progressed.

**FIGURE 1:**
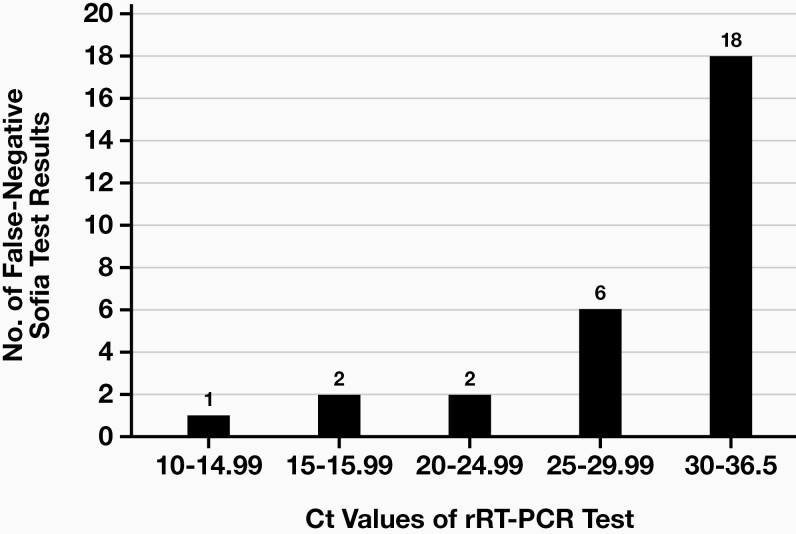
Presentation of the false-negative results in testing with the Sofia test with respect to the Ct values of the rRT-PCR test in group 2. Sofia, Sofia SARS Antigen Fluorescent Immunoassay; rRT-PCR, real-time reverse transcription–polymerase chain reaction; Ct, cycle threshold.

In group 2, we observed a higher number of false-negative results. These results are presented in **FIGURE 1**. In this figure, a significantly higher number of false negative values at higher Ct values can be observed. Nevertheless, these values are not extremely low, as the rRT-PCR test classifies Ct values below 29 as strongly positive or having high concentrations of the target nucleic acids, values from 30 to 37 as moderate amounts of the target nucleic acids, and values from 38 to 40 as extremely low amounts, which could also represent a state of infection or contamination of the sample.^12^

It is important to note that there were only 7 truly positive Sofia test results at Ct values greater than 30, as opposed to the 18 false-negative results. We can therefore conclude that the Sofia test only detects very high concentrations of the virus in the sample reliably (Ct values 10–25), whereas at low concentrations, it is significantly less reliable. However, the Ct values must be interpreted carefully as they are affected by sample type, sample collection timing, and assay design.^13^

### Statistical Comparison of the Diagnostic Accuracy of the Tests

To compare the diagnostic accuracy of the Sofia test with the rRT-PCR test, we first performed the McNemar test. The result of the 2-way test in group 1 was 0.625. Since this value is higher than 0.05, we cannot reject the null hypothesis. Thus, there was no statistically significant difference between the Sofia and rRT-PCR tests in group 1. However, the result of the 2-way test in group 2 was less than 0.05, so we can reject the null hypothesis and accept the alternative hypothesis. Therefore, a statistically significant difference can be observed between the 2 tests in group 2.

Cohen’s Kappa test was also performed for comparison purposes. The Kappa value in group 1 was 0.802, which shows a strong correspondence between the Sofia test and the rRT-PCR test. Additionally, our result confirms the correctness of the McNemar test result. The same analysis was performed for group 2. The Kappa value in group 2 was also higher than 0.8, which indicates a strong agreement of the tests. Using the McNemar test, however, we proved a disagreement in the case of group 2, which, according to the data used by the test for analysis, indicates a low level of disagreement.

It can be concluded that the clinical sensitivity and specificity of the Sofia test in the case of symptomatic subject testing are comparable to the values of the rRT-PCR test. However, in the case of testing both asymptomatic and symptomatic patients, the Sofia test is clinically less reliable than the rRT-PCR test in terms of the number of false-positive results.

As already observed with analytical sensitivity, most false-negative results occurred in samples where Ct values were higher than 30. We were interested in the extent to which these results affect the statistical comparability of tests. For this purpose, we also performed a statistical comparison of the results of group 2, which did not include the results of samples with Ct values higher than 30.

The results of both the McNemar test (p = .332) and the Kappa test (K = 0.914) show a strong correspondence between the methods. We can therefore conclude that the Sofia test is comparable to the rRT-PCR method in the case of testing samples with high analyte concentration and significantly less reliable at lower concentrations (Ct > 30). This can pose an obstacle, especially when testing patients in the early stages of infection when the virus concentration in the sample may be low.

Sensitivity and specificity determine the operational characteristics of the test, but the predictive value (positive or negative) of the test is of great diagnostic importance to the physician and patient.^14^ In **TABLES 3** and **4**, it can be observed that in group 1, the probability that a person with a positive test result has the disease is 75.0% (the probability of a person with a negative result not having the disease is 99.2%). Negative results can therefore be trusted in group 1, but regarding a positive result, there is a 25.0% probability that the positive result does not show the presence of an actual disease. This result would not be favorable in a disease where confirmatory tests are invasive or may even worsen the patient’s health. In the case of COVID-19, all patients with a positive Sofia test can be tested with the rRT-PCR test to confirm their infection. Significantly more important in SARS-CoV-2 infection is a good negative prognostic value, as any patient with a negative result that is actually positive remains unrecognized and consequently unknowingly spreads the infection. The negative predictive value in group 1 was 99.2% and 95.0% in group 2.

Positive and negative predictive values also depend, among other things, on the prevalence of the disease in the tested population.^15^ This is the reason for the low positive predictive value in group 1, although the sensitivity and specificity values are high. However, it should also be emphasized that the prevalence of the disease differs between the 2 groups as the sampling period was completely different.

## Discussion

The purpose of the study was to compare the Sofia test with the rRT-PCR test intended for the detection of SARS-CoV-2 virus. During the analysis of the data, we showed that there is no statistically significant difference in the diagnostic accuracy between the Sofia test and the rRT-PCR test when testing symptomatic subjects. However, the same is not true for asymptomatic persons. The concentration of the virus in the sample had a significant effect on the efficiency of the test, as we observed a significantly higher number of false-negative results in the samples with higher Ct values. The tests also differ in the way they are performed. The Sofia test is designed to be performed as POCT.^5^ Its implementation is therefore less demanding and does not require specially trained staff. It can be performed by medical staff to whom the method and its proper implementation have been presented by an expert. The correct performance of the rRT-PCR test, however, is much more demanding and requires a high level of accuracy and precision of trained staff.^16^ It should also be emphasized that the rRT-PCR test is significantly more expensive and time-consuming than the Sofia test due to its complexity.

Fundamentally, the analytical specificity of both methods is high. Antibodies to immune methods are capable of very specific recognition of a particular antigen.^17^ The analytical specificity of PCR methods is based on the fact that specific nucleotide sequences can be determined in the viral RNA sequence, and specific oligonucleotide primers can be designed accordingly.^18^ The analytical specificity of the Sofia test was not specifically defined by the manufacturers, nor could we define it in our research. However, we can compare data from the literature on the cross-reactivity of both tests. The cross-reactivity of the Sofia test was assessed by the manufacturer by testing various microbes, 16 viruses, and 3 negative matrices. All viruses and microbes were tested in the presence and absence of heat-inactivated SARS-CoV-2. The manufacturers demonstrated the absence of cross-reactivity with all tested microbes.

We can also infer the robustness of the methods. The robustness of the rRT-PCR assay stems from the fact that oligonucleotide primers are capable of close and specific binding to complementary nucleic acid sequences. The method itself is sensitive to contamination and the presence of inhibitors, but good optimization of the method significantly improves its robustness.^16^ Automation can also help increase the robustness of the method. The Sofia test is intended for use with a patient, so it is important for the success of the test and the reliability of its results that the method is very robust. The device is portable and analysis can be performed by suitably qualified medical personnel. In the case of the Sofia test, the antibodies of the immune test are capable of close and specific binding to antigens, which significantly contributes to the robustness of the method itself.

The pitfall of both tests is that they are no longer reliable after the concentration of viral RNA and viral antigens falls below the detection limit.^19^ The severity of the disease, the timing of sample collection, the types of sample, and sample handling techniques all influence antigen levels in samples.^13^ The results discussed in this article were obtained as part of the testing of patients who were brought to 2 different departments of the University Medical Centre Ljubljana. Due to the nature of the testing, sampling and analysis were performed by several different individuals, so the possibility of errors in sample handling cannot be completely ruled out. We also do not possess the information on antigen levels in the samples; therefore, it is also hard to determine whether the difference in observed sensitivity is due to the test performance or the qualities of the samples used in the test.

Despite its shortcomings, rRT-PCR is still considered the gold standard for the diagnosis of SARS-CoV 2. Sources, however, point to shortcomings in diagnostics that rely solely on the detection of nucleic acids, mainly the large inconsistencies and a high rate of false-negative values. As a solution, they suggest combining testing with imaging of thoracic organs and other clinical signs.^19^ We wondered whether combining the Sofia test with the rRT-PCR test would reduce these shortcomings. Due to the low detection limit of the Sofia test, combining these 2 methods would probably not significantly help to improve these deficiencies; however, the Sofia test can serve as a rapid screening test, as it can be performed at the point of care in significantly less time than rRT-PCR.

Our results therefore show that the Sofia test meets the standards of a reliable screening test according to the World Health Organization criteria. It shows high sensitivity and specificity, and perhaps most importantly, a high negative predictive value. This study confirms that the Sofia test can be used as a screening test, especially in circumstances that require rapid treatment and triage of patients, as the test can be quickly carried out at the point of care.

## Abbreviations

Sofia: Sofia SARS Antigen Fluorescent Immunoassay
rRT-PCR: real-time reverse transcription–polymerase chain reaction
FIA: fluorescent immunoassay
POCT: point-of-care testing
Ct: cycle threshold
